# Compartment specific regulation of sleep by mushroom body requires GABA and dopaminergic signaling

**DOI:** 10.1038/s41598-021-99531-2

**Published:** 2021-10-08

**Authors:** Margaret Driscoll, Steven N Buchert, Victoria Coleman, Morgan McLaughlin, Amanda Nguyen, Divya Sitaraman

**Affiliations:** 1 Department of Psychological Sciences, College of Arts and Sciences, University of San Diego, 5998 Alcala Park, San Diego, CA 92110 USA; 2 Department of Psychology, College of Science, California State University- East Bay, 25800 Carlos Bee Blvd, Hayward, CA 94542 USA

**Keywords:** Neuroscience, Circadian rhythms and sleep, Neural circuits, Genetics, Behavioural genetics

## Abstract

Sleep is a fundamental behavioral state important for survival and is universal in animals with sufficiently complex nervous systems. As a highly conserved neurobehavioral state, sleep has been described in species ranging from jellyfish to humans. Biogenic amines like dopamine, serotonin and norepinephrine have been shown to be critical for sleep regulation across species but the precise circuit mechanisms underlying how amines control persistence of sleep, arousal and wakefulness remain unclear. The fruit fly, *Drosophila melanogaster*, provides a powerful model system for the study of sleep and circuit mechanisms underlying state transitions and persistence of states to meet the organisms motivational and cognitive needs. In *Drosophila*, two neuropils in the central brain, the mushroom body (MB) and the central complex (CX) have been shown to influence sleep homeostasis and receive aminergic neuromodulator input critical to sleep–wake switch. Dopamine neurons (DANs) are prevalent neuromodulator inputs to the MB but the mechanisms by which they interact with and regulate sleep- and wake-promoting neurons within MB are unknown. Here we investigate the role of subsets of PAM-DANs that signal wakefulness and project to wake-promoting compartments of the MB. We find that PAM-DANs are GABA responsive and require GABA_A_-Rdl receptor in regulating sleep. In mapping the pathways downstream of PAM neurons innervating γ5 and β′2 MB compartments we find that wakefulness is regulated by both DopR1 and DopR2 receptors in downstream Kenyon cells (KCs) and mushroom body output neurons (MBONs). Taken together, we have identified and characterized a dopamine modulated sleep microcircuit within the mushroom body that has previously been shown to convey information about positive and negative valence critical for memory formation. These studies will pave way for understanding how flies balance sleep, wakefulness and arousal.

## Introduction

Sleep, wakefulness and arousal represent internal states that control multiple physiological and behavioral processes. Transition between these states and persistence of these individual states involves neural circuits that are dispersed throughout the brain and interact with systems involved in controlling hunger, goal-directed behavior, and memory formation.

While, sleep provides many benefits, it limits the ability of the organism to engage in other behaviors critical for survival. Sleep and wakefulness are actively balanced and influenced by motivational or cognitive processes that modulate transition and persistence of these states^1,2^. Hence, there are additional processes that regulate sleep and are strongly influenced by conflicting needs (internal and external) which are not accurately represented in the existing models.

At the behavioral level motor output, attention, motivation, reward, and feeding function on the basis of wakefulness and represent different levels of arousal and involve conserved neuromodulators like dopamine^3–5^, serotonin^6–9^ and histamine^10–13^. Neuromodulators are highly conserved across species and can target synapses by altering excitability of neurons that generate variable output from defined circuits^14–16^. Hence, a comprehensive understanding of how neuromodulators influence structures involved in sleep, wakefulness and other associated behaviors can provide an inroad into understanding the highly plastic nature of sleep regulation.

Dopamine is a wake-inducing neuromodulator in flies and mammals^17–21^. The ability to manipulate genes and genetically defined neural circuits has allowed identification of distinct sleep–wake microcircuits and their modulation by dopamine in flies and rodents^1,2,17–19,22–25^. Broad manipulations of DANs using genetic and pharmacological approaches shows that dopamine is required for wakefulness and this is further supported by altered sleep in receptor and transporters mutants^22,25^.

In the most well-defined dopamine-mediated wake circuit in flies, a cluster of PPL1 and PPM3 neurons project to the central complex innervating the dorsal fan-shaped body region (dfb) that has been shown to encode sleep need and critical for sleep homeostasis^20,21^. Specifically, the dorsal fan shaped body dependent sleep switch is inhibited by dopamine input via DopR2 signaling and altered potassium conductance^23,26,27^. Artificial PPL1 cluster activation or direct dopamine application electrically silence sleep-promoting dfb neurons by altering the receptivity of the dopamine arousal signal, but it is unclear if the PPL1 neurons themselves signal sleep need^23,26,27^. While, DA dependent mechanisms of sleep-regulation have been identified within the CX^26^ and circadian clock system^28,29^, the widely used TH-GAL4 excludes several PAM neurons which provide key DA inputs to Mushroom Body (MB)^30^.

Thermogenetic activation of DANs projecting to regions outside CX, specifically that innervate the MB are also wake-promoting, suggesting that dopamine clusters induce wakefulness via distinct neural structures^31,32^. Two separate clusters of neurons called PAM and PPL (~ 130–150 neurons) account for the majority of dopamine signaling in the fly brain and represent the most extensive neuromodulator input to the MB, a key center required for associative learning in insects^33,34^. MB is a lobed structure where 2000 Kenyon cells send out parallel axonal fibers that form two vertical and three horizontal lobes with dendrites organized within the calyx. 22 MBONs (MB Output neurons) innervate the lobes and receive input from the KCs and form distinct compartments. Each of these compartments, receives modulatory input from one or more of the 20 subsets of dopaminergic neurons (DANs)^32,35–39^. The core KC-MBON circuits are modulated by DANs, which signal olfactory cues, satiety, wakefulness, negative and positive valence, and novelty/familiarity of stimuli^39–45^. But there is growing evidence that DANs signal a wide-range of information to the MB about novelty^39^, satiety^40^, locomotion^46,47^ and sleep/activity states^32,48^. Furthermore, there is neuroanatomical, physiological, and biochemical evidence that DANs adjust and tune synaptic weights between KCs and MBONs, across multiple compartments but the mechanisms related to receptors and downstream signaling are not uniform^49–51^.

In our previous work, we comprehensively identified the KCs, MBONs, and DANs that control sleep, by performing an unbiased thermogenetic activation screen using a new library of intersectional split-GAL4 lines^36,52^. We identified several classes of sleep-controlling MBONs with dendrites in distinct lobe compartments: cholinergic sleep-promoting MBON-γ2α′1, and glutamatergic wake-promoting MBON-γ5β′2a/β′2mp/β′2mp_bilateral and MBON-γ4 > γ1γ2. The sleep effects were consistent in both males and females and we did not find any sexually dimorphic sleep phenotypes or neuroanatomical differences in GFP expression^31,52^. We also determined that α′/β′ and γm KCs are wake-promoting and γd KCs are sleep-promoting, and that α′/β′ and γm KCs promote wake by activating MBON-γ5β′2 and γd KCs promote sleep by activating MBON-γ2α′1^31,52^.

Each of these sleep-regulating compartments are also innervated by DANs. PAM neurons specifically project to the β, β′, α1, and γ lobes, while PPL1 neurons innervate the vertical lobes (α and α′), heel, and peduncle, and PPL2ab neurons project to the calyx^30^. In an unbiased screen of all DANs projecting to the MB using a cell specific split-GAL4 library, thermogenetic activation using the dTRPA1 revealed that multiple classes of PAM and PPL1 DANs suppress sleep^31,32^. PAM-DANs that project to wake-promoting α′/β′ KCs, γm KCs and MBON-γ5β′2 are strongly wake promoting and transient P2X2 mediated activation of PAM DANs induces robust transient Ca^2+^ increases in these MB compartments. The Ca^2+^ signal was completely blocked by bath application of the competitive D1-specific dopamine receptor antagonist SCH23390 suggesting that dopamine acts on KCs and MBONs via D1 receptors^32^.

In addition to dopamine, GABA signaling also modulates sleep and wake microcircuits within MB^53^. The key source of GABA in the MB is anterior paired lateral neurons, APL and dorsal paired medial neurons (DPM), which are electrically coupled and increase sleep by GABAergic inhibition of wake-promoting KCs^53^. In the context of associative learning, there is strong evidence for interactions between KCs, APL and DANs^54,55^ but it is not clear if GABA and dopamine signaling represent opposing inputs to the KCs and MBONs in the regulation of sleep.

Here we identify the circuit, cellular, and molecular basis of how subsets of PAM DANs regulate wakefulness and test potential interactions between DA and GABA signaling. Specifically, we report that PAM DANs projecting to γ5, γ4 and β′2 MB compartments are GABA responsive and regulate sleep via the ionotropic GABA_A_-Rdl receptor. Furthermore, we show that the wake promoting effects of PAM-DANs projecting to γ5 and β′2 MB compartments are mediated by two of the four dopamine receptors, DopR1 and DopR2, which function within the KCs and MBONs of wake-regulating MB compartments.

The PAM-DANs projecting to γ5, γ4 and β′2 MB compartments have been previously implicated in context-dependent arousal associated with sugar reward and electric shock used to reinforce memories in a cell-type specific manner^37,38,41^. Therefore, it is likely that differential synaptic inputs and modes of communication to KC-MBONs via these DANs modulates MB function in promoting sleep, wakefulness, or arousal induced by cues associated with learning and memory formation.

## Results

### PAM-DANs are GABA responsive and require Rdl activity to regulate sleep

In an unbiased screen of all DANs projecting to the MB using a cell specific split-GAL4 library, thermogenetic activation using the dTRPA1 temperature-gated depolarizing cation channel revealed that multiple classes of PAM DANs suppress sleep^31,32^. Of these cell classes PAM DANs projecting to γ5, γ4 and β′2 MB compartments had the strongest phenotypes^32^. In addition to DANs, GABA signaling to these MB compartments promotes sleep^53^. While, GABAergic input has shown to inhibit activity of KCs^56^ within these compartments, it is not clear if they directly or indirectly influence the activity of PAM DANs.

We tested if activity of the DANs projecting to γ5, γ4 and β′2 MB compartments is altered by GABA. To this end, we expressed ATP-gated cation channel P2X_2_ along with calcium sensor GCamp6m in PAM DANs labelled by MB196B that targets the γ5, γ4, β′2 and β2 MB compartments. Activation of MB196B by expression of dTrpA1 induces wakefulness and inhibition of these neurons promotes sleep^32^.

**Figure 1:**
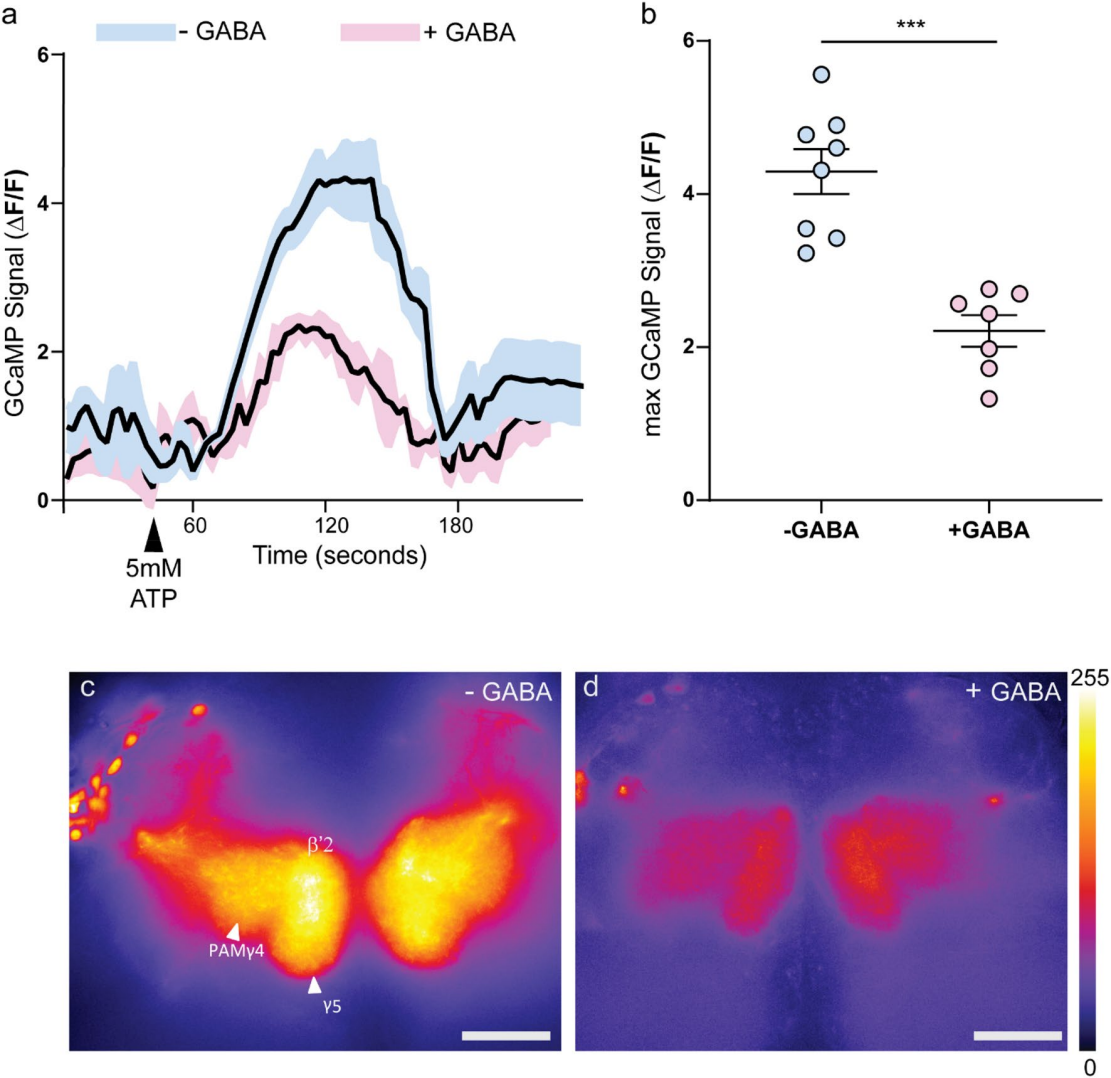
PAM-DANs required for wakefulness are GABA responsive. An ATP-gated cation channel P2X2 was expressed in PAM neurons using MB196B split-GAL4 line along with a calcium sensor GCaMP6m (196B > P2X2, GCaMP6m) to quantify ATP-induced changes in intracellular Ca^2+^ levels as a readout of the neural excitability in presence and absence of GABA. Whole brains were dissected and imaged in HL3 buffer. (**a**) Time series of fluorescence images recorded to measure Ca^2+^ signals. PAM-DANs were activated by bath application of 5 mM ATP (black arrow indicates ATP application). Brains were incubated with 50 mM GABA (+ GABA, pink) or in HL3 (−GABA, blue) 5 min prior to recording. Data represents mean and SEM (n = 7–8) of ΔF/F = (Ft − Fo)/Fo) where Fo = is defined as the average background subtracted baseline fluorescence for the ten frames preceding ATP application. (**b**) Maximum ΔF/F in the presence (pink) and absence of GABA (blue). Two conditions were compared by Unpaired t-test with Welch’s correction, two-tailed p value was < 0.0001. (**c**,**d**) Representative images indicating ROIs (PAM-DAN innervations in γ5, β′2, and γ4 of MB lobes) used for comparison for Ca^2+^ signals. Left panel (−GABA or no GABA) and right panel indicated recording in the presence of GABA. Scale bar indicated 10 μm and scale of fire LUT used to emphasize pixel intensity (0–255) is shown for reference.

We found that bath application of 5 mM ATP to whole brain explants imaged in HL3 solution induces a robust increase in fluorescence signal indicative of elevation of intracellular calcium levels. However, pre-incubation of whole brain explants in 50 mM GABA for 5 min prior to recording and ATP application suppressed the excitability to PAM DANs (Fig. 1a). The ROI included γ5, γ4, β′2, and β2 regions (Fig. 1c,d) and ΔF/F indicative of maximum GCaMP signal (Fig. 1b) in brain explants was significantly different in the presence and absence of GABA.

Although, our results show that PAM DANs are GABA responsive it is not clear if the inhibitory effects are direct or indirect (likely mediated by KCs). To address if the GABA inhibition is direct or indirect and critical for PAM DAN mediated sleep regulation, we examined if RNA interference mediated depletion or downregulation of metabotropic and ionotropic GABA receptors in PAM DANs causes changes in sleep phenotypes.

Specifically, we tested if reducing expression of GABA receptors in MB196B influenced sleep. We targeted the ionotropic GABA_A_ type receptor (Rdl)^57,58^ and 3 metabotropic GABA_B_ receptors (GABA_B_ R1, GABA_B_ R2, and GABA_B_ R3)^59^ using validated UAS-RNAi lines^53,60^. To determine if these receptors regulate sleep, we measured sleep in MB196B expressing UAS-RNAi lines. A negative control “empty” split-GAL4 line was used as control that lacks active genomic enhancer sequences and has the same genetic background as MB196B.

**Figure 2:**
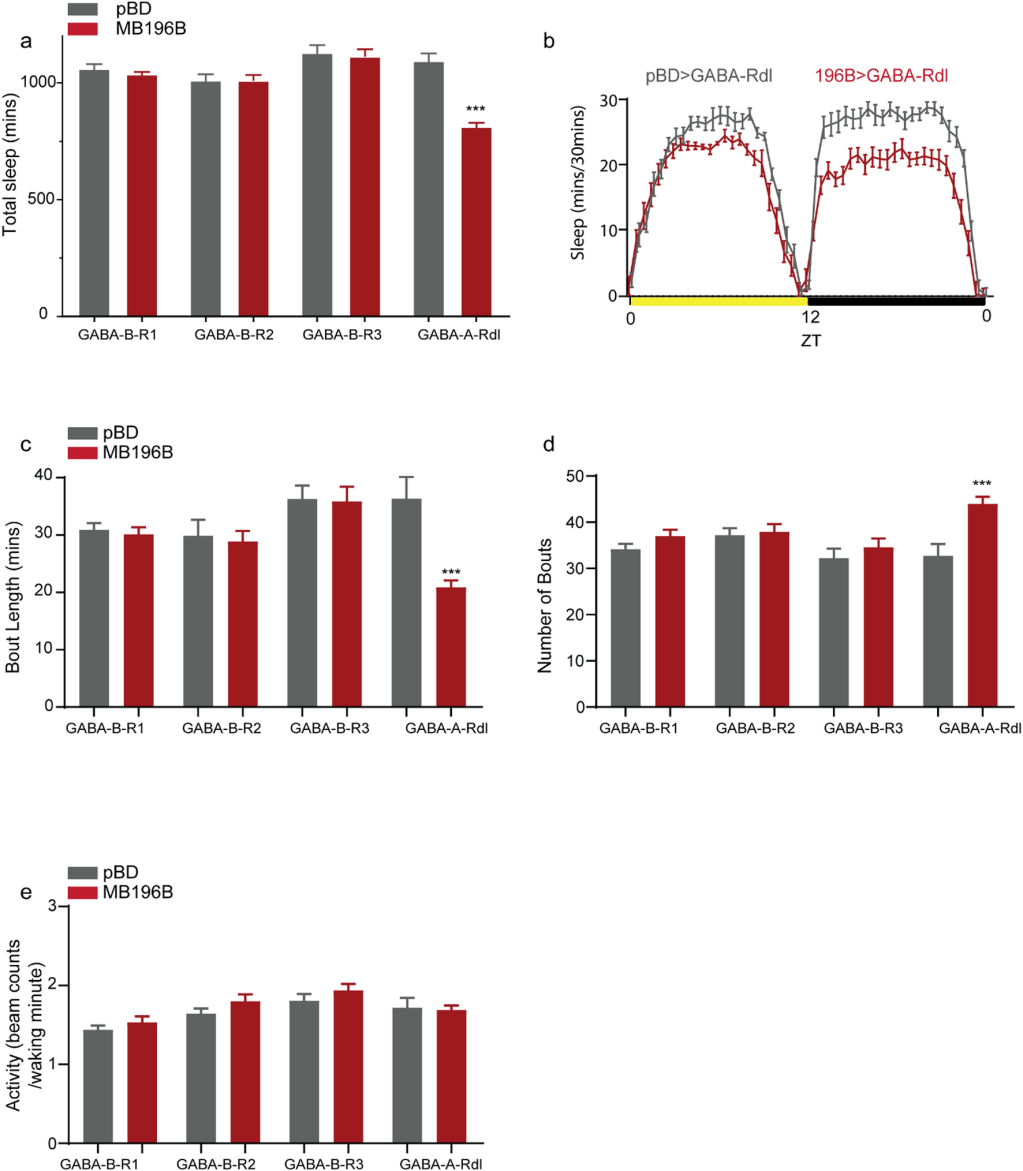
PAM-DAN mediated wakefulness is inhibited by GABA and GABA_A_ Rdl receptor expression. RNAi lines targeting GABA_A_ and GABA_B_ receptors were expressed in PAM-DANs targeted by MB196B or control (empty split-GAL4/pBD) and sleep was measured using Drosophila Activity Monitoring system. Sleep data represents 2-day average after 2-days of 12 h light and 12 h. dark entrainment. (**a**) Total sleep or sleep duration over 24 h in flies expressing validated RNAi lines targeting one of the GABA_B_ (R1, R2 and R3) or GABA_A_ (Rdl-Resistance to dieldrin) receptor or subunits in wake-promoting PAM-DANs (MB196B: red) and enhancerless/empty-GAL4 (pBD: grey) in the same genetic background. Significant differences were detected between MB196B and pBD driver lines expressing UAS-RNAi targeting GABA_A_ Rdl receptor. (**b**) Representative sleep profile of RNAi mediated depletion of ionotropic GABA_A_ Rdl receptors in PAM MB196B (red) and empty/enhancerless-GAL4 control (grey). ZT indicates zeitgeber time where ZT 0: lights on and ZT 12: lights off. (**c**,**d**) Average bout length (minutes) and average number of bouts. Significant differences were detected between MB196B and pBD driver lines expressing UAS-RNAi targeting GABA_A_ Rdl receptor. Significant differences were detected between MB196B and pBD driver lines expressing UAS-RNAi targeting GABA_A_ Rdl receptor. (**e**) Activity or average beam crossings/waking minute indicative of locomotor activity of all tested genotypes. No significant differences were detected. For each of the experimental groups we had 34–41 flies which represents 2 independent experimental trials. Number of flies for each genotype were: GABA_B_-R1 (MB196B: 37, pBD: 40), GABA_B_-R2 (MB196B: 39, pBD: 41), GABA_B_-R3 (MB196B: 35, pBD: 38), and GABA_A_-Rdl (MB196B: 34, pBD:36). In this and all subsequent figures data represents mean and SEM, * indicates p < 0.05, ** indicates p < 0.001 and *** indicates p < 0.0001. Statistical analysis was Mann–Whitney U-test post-hoc analysis between MB196B and control expressing the same UAS-RNAi lines for (**a**), (**c**), (**d**), and (**e**).

We found that total sleep (sleep over 24 h represented as 2-day average, Fig. 2a,b) measured using the Drosophila Activity Monitoring system^61^, was significantly reduced in transgenic flies where GABA_A_ type receptor (Rdl) was knocked down. We did not find any changes in sleep by knockdown of metabotropic GABA_B_ receptors. Further, analysis of the sleep phenotype shows that Rdl knockdown specifically decreases sleep bout length (Fig. 2c) and increases number of sleep bouts (Fig. 2d) as compared to control. The sleep suppression or wakefulness induced by depletion of Rdl receptor did not affect locomotor activity and activity was measured as number of beam crossings per waking minute (Fig. 2e).

**Figure 3:**
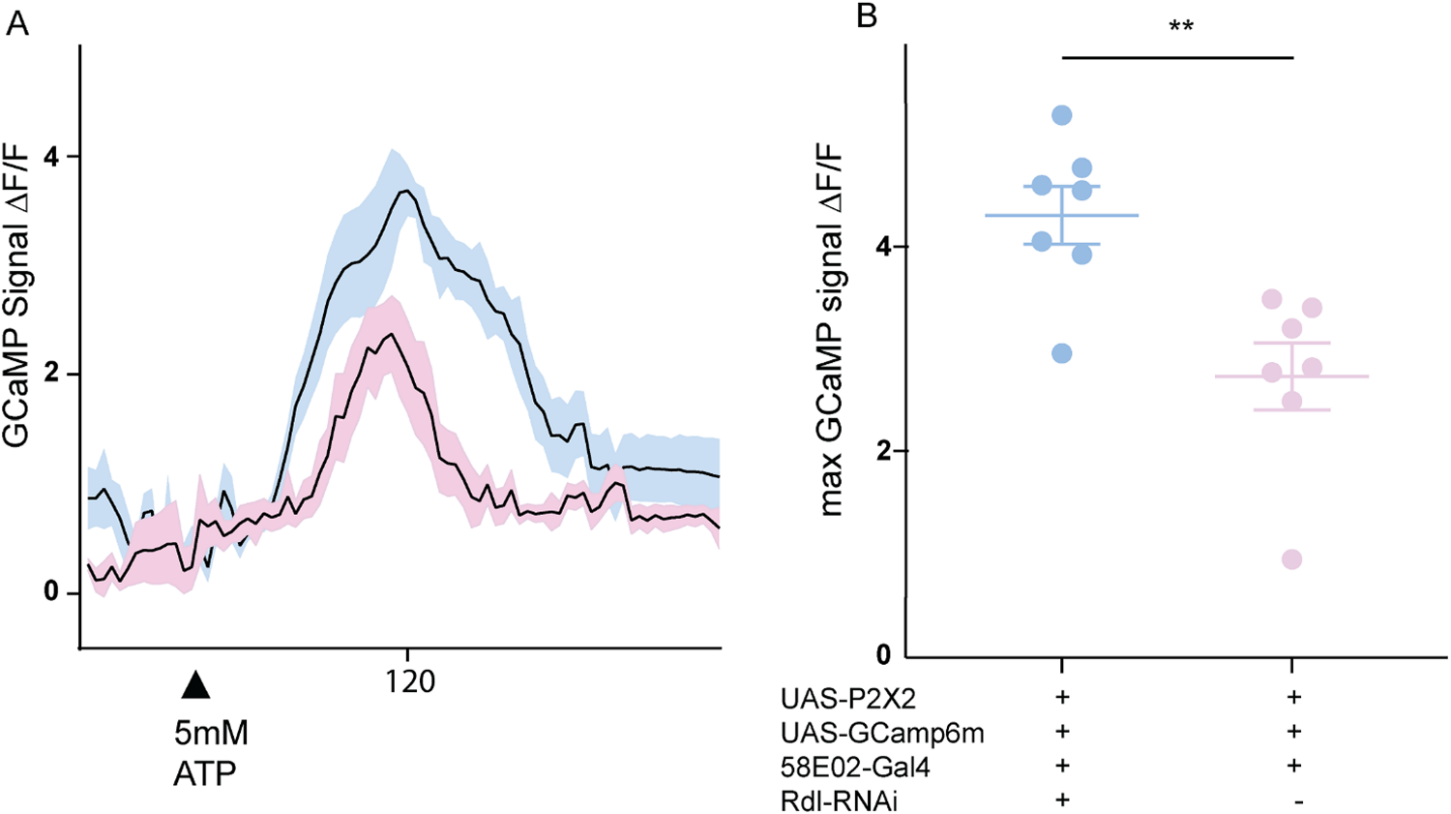
PAM-DANs required for wakefulness are GABA responsive and require Rdl receptors. An ATP-gated cation channel UAS-P2X2 was expressed in PAM neurons using 58E02-GAL4 line along with a calcium sensor UAS-GCaMP6m and UAS-Rdl-RNAi to quantify ATP-induced changes in intracellular Ca^2+^ levels in the presence of GABA. All imaging conditions including ROI selection and drug treatment were consistent between samples. (**A**) Time series of fluorescence images recorded to measure Ca^2+^  signals. PAM-DANs were activated by bath application of 5 mM ATP (black arrow indicates ATP application) in flies with (blue) or without (pink) UAS-Rdl-RNAi transgene. In both cases fly brains were incubated with 50 mM GABA. Data represents mean and SEM (n = 7) of ΔF/F = (Ft − Fo)/Fo) where Fo = is defined as the average background subtracted baseline fluorescence for the ten frames preceding ATP application. (**B**) Maximum or Peak ΔF/F was used for statistical analysis to quantify GABA induced decrease in PAM excitability in the presence (blue) and absence (pink) of UAS-Rdl-RNAi. Two conditions were compared by Unpaired t-test with Welch’s correction, two-tailed p value = 0.0035.

To better understand the physiological significance of GABAergic signaling to PAM neurons and a potential role of Rdl we conducted Ca^2+^ imaging experiments in PAM neurons targeted by 58E02-Gal4 that co-expressed UAS-Rdl RNAi. Specifically, we asked if the observed decrease in PAM activity evoked by mis-expression of P2X2 and ATP application in the presence of GABA (Fig. 1) requires Rdl function. We found that in flies where GABA_A_ Rdl receptors were knocked down, the decrease in PAM excitability induced by GABA was suppressed (Fig. 3). These data show that GABA induced suppression of PAM excitability requires Rdl receptor expression.

Taken together, our behavioral and physiological data show that subsets of PAM DANs that activate the wake-regulating compartments of MB and induce wakefulness are inhibited by GABA signaling and the effects of GABA are mediated by ionotropic GABA_A_ type receptor Rdl.

### PAM-DAN signaling to specific MB compartments is required for sleep regulation by GABA signaling

The role of GABA and Rdl receptor has been previously shown to be important for sleep regulation. Carbamazepine or CBZ is a pharmacological agent that reduces GABAergic transmission by accelerating the desensitization of Rdl, Resistance to Dieldrin (GABA_A_ ionotropic receptor), and shown to suppress total sleep and increase sleep latency in a dose-dependent manner^62^. Further, *Rdl*^*MDRR*^ mutants have enhanced GABAergic transmission due to altered channel properties of the Rdl receptors and exhibit shorter sleep latency and increased sleep^62^. While, the CBZ and Rdl effects on sleep are thought to be modulated by Pdf neurons^62,63^, the gene *Rdl* is expressed at high levels in the MB lobes and MBONs^40,64^. Our calcium imaging and Rdl knock down experiments show that GABA signaling inhibits excitability of PAM DANs and are required for wakefulness via Rdl expression in MB196B that targets multiple PAM-DAN subsets.

To identify subsets within MB196B relevant to GABA and Rdl signaling, we silenced smaller subsets of dopaminergic neurons using restricted split-Gal4 drivers to express the temperature-sensitive dynamin mutant Shibire^ts1^ (Shi^ts1^) in the presence of CBZ.

We used six restricted split-GAL4 lines including two broader PAM lines (MB196B: PAM γ5, γ4, γ4 > γ1,2, β′2a, β2a and MB194B: PAM γ5, β1, β′1, α1) and four narrow PAM lines (MB054B: PAM γ5, MB312B: PAM γ4 + PAM γ4 > γ1,2, MB213B: β1 + β2, and MB209B: β2 + α1). These split-GAL4 lines are wake-promoting and induced wakefulness when transiently activated by dTrpA1 expression^32^. Expression patterns within MB compartments was confirmed by GFP expression and are shown in Figure S1.

**Figure 4:**
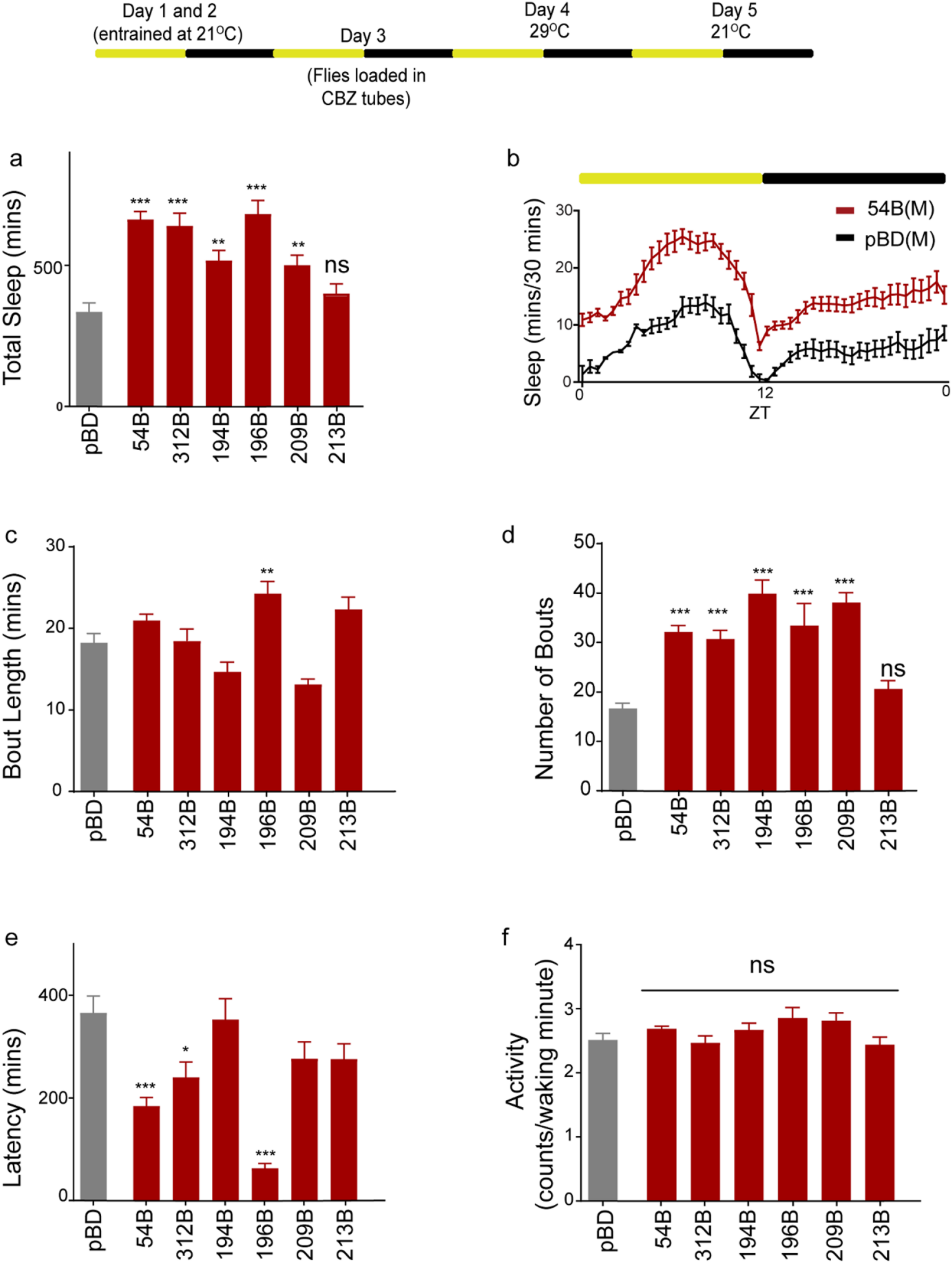
Wakefulness induced by pharmacologic suppression of GABA_A_ receptor, Rdl requires specific subsets of MB DANs. Schematic of experimental protocol showing temperature and drug conditions over a 5-day period. Flies were entrained for Day 1 and 2 in vials. Following entrainment flies were loaded on CBZ containing food (Day 3) in incubator maintained at 21 °C. Day 4 the temperature was switched to 29 °C and used for quantification and analysis shown below. (**a**) Total sleep in PAM-DAN subsets (red) labelled by split-GAL4 lines, MB054B, MB312B, MB194B, MB196B, MB209B, and MB213B where neural activity has been suppressed by over-expressing temperature sensitive dominant negative dynamin mutation, Shi^ts1^ in the presence of CBZ (Day 4). Enhancerless/empty-GAL4 in the same genetic background as PAM split-GAL4 lines was used as control (grey). (**b**) Representative sleep profile of flies on Day 4 with targeted inhibition of specific PAM MB054B (red) and empty/enhancerless-GAL4 control (black). ZT indicates zeitgeber time where ZT 0: lights on and ZT 12: lights off. (**c**,**d**) Average bout length (minutes) and average number of bouts on day 4. (**e**) Sleep latency or time to sleep from lights off (ZT 12) was calculated as the time gap in minutes between lights off and first sleep bout. (**f**) Activity or average beam crossings/waking minute indicative of locomotor activity of all tested genotypes. For each of the experimental groups we had 73–98 flies which represents 4 independent experimental trials. Number of flies for each genotype were: pBD (n = 96), 54B (n = 98), 194B (n = 77), 209B (n = 81), 213B (n = 73), 312B (n = 82), and 196B (n = 75). In this and all subsequent figures data represents mean and SEM, * indicates p < 0.05, ** indicates p < 0.001 and *** indicates p < 0.0001. Statistical analysis was one-way ANOVA and Dunnett’s paired comparison with control for (**a**,**e**,**f**) and Kruskal–Wallis non-parametric one-way ANOVA and Dunn’s post-hoc correction for (**c**,**d**).

At the restrictive temperature, 29°C, the targeted neurons have blocked synaptic transmission^65^. Only one of the split-GAL4 lines MB213B still permitted CBZ induced loss of sleep at 29 °C when driving Shi^ts^ as compared to control. Five split-GAL4 lines (MB054B, MB312B, MB 196B, MB194B, and MB209B) did not show CBZ-induced sleep decrease when driving Shi^ts1^ at 29 °C (Fig. 4a,b). The experimental groups were compared to negative control “empty” split-GAL4 line that lacks active genomic enhancer sequences and has the same genetic background as the other split-GAL4 lines. Hence, we find that inhibition of multiple subsets of PAM-DANs, oppose the wake-promoting effects of CBZ.

In addition to blocking the sleep suppression phenotype of CBZ, inhibition of all five split-GAL4 lines (MB054B, MB312B, MB 196B, MB194B, and MB209B) increased the number of sleep bouts (Fig. 4d). Only MB196B inhibition increased the average length of sleep bout (Fig. 4c). We next looked at how altering activity of PAM DANs influenced CBZ induced latency. Like total sleep phenotype, latency was reduced strongly in multiple PAM drivers (MB054B, 312B and 196B) as compared to control (Fig. 4e). Activity measured as beam crossings/waking minute were consistent between genotypes (Fig. 4f) suggesting that the genetic manipulations and temperature elevation did not have differential effects on locomotor activity of the tested genotypes.

Although, MB054B is a strong driver of PAM γ5, it also targets PAM γ3. To address a more specific role for MB054B we repeated these experiments with MB315B, a cleaner split-GAL4 driver of PAM γ5 and MB441B that specifically targets PAM γ3 (Figure S2). We also ran additional genotypic controls (MB054B/+, MB315B/+, MB441B/+ and Shi^ts1^/+) at restrictive temperature which supports the finding that specific PAM-DANs are wake-promoting in the presence of CBZ (Figure S2).

To test if genotypes used in the study had differential sensitivity to CBZ and that these effects are specific to Shi^ts1^ based inhibition we measured sleep in the presence of CBZ at 21 °C (permissive temperature). Flies expressing Shi^ts1^ in PAM-DANs (54B, 312B, 196B, 194B, 209B and 213B) had reduced total sleep (~ 550–600 min) at permissive temperature and were not significantly different from controls (Figure S3a,b). Activity and latency were not significantly different between all tested genotypes (Figure S3c,d). These data indicate specific PAM DANs expressing Shi^ts1^ suppress CBZ induced wakefulness as compared to genotypic controls at restrictive temperature. CBZ induced wakefulness is consistent between PAM DANs expressing Shi^ts1^ and not significantly different from controls at permissive temperature.

While the effects of CBZ on sleep in flies are thought to be specific to GABAergic modulation of the Pdf neurons^62^, our data shows that CBZ effect on sleep is regulated in part by MB. This is supported by the abundance of Rdl receptors in MB and their role in regulating calcium dynamics within MB lobes^64,66^. GABA and dopamine have known to work antagonistically within MB in regulating sleep and our data shows that CBZ induced  wakefulness can be suppressed by blocking dopamine release to specific MB compartments. We also find that release of dopamine from PAM γ5, γ4 and β′2a (MB054B, MB315B, MB312B, and MB196B) had the stronger effects on CBZ induced wakefulness as compared to MB194B and MB209B that label β2, β′1, β1 and α1 compartments. Like PAM-DANs (MB054B, MB312B and MB196B) synaptic silencing of the downstream wake-promoting MBONs in the γ5, γ4 and β′2 compartments suppress the wake-promoting effects of CBZ^31,32,36,52^.

To address the downstream pathways from PAM DANs innervating γ5, γ4 and β′2a MB compartments we focussed our attention on the role of dopamine receptors in sleep regulation within the MB.

### Dopamine signals wakefulness via DopR1 and DopR2

Four dopamine receptors (all G-protein coupled receptors) have been identified in the Drosophila genome: DopR1, DopR2, D2R and DopEcR^67–70^. As in humans, DopR1 and DopR2 are D1-like receptors and functions via activation of the cAMP pathway, while D2-like receptors inhibit this pathway. Hence, the effect of DA on a specific postsynaptic neuron depends on the type of DA receptor that is expressed. Dopamine receptors DopR1 and DopR2 are highly expressed in the MB (KCs and MBONs) and have been shown to increase production of cAMP in in-vitro assays^37,68,71,72^.

DA receptor or transporter mutations have been shown to increase arousal thresholds (to air puffs, light or mechanical stimuli) in awake flies, independent of their role in sleep^22,73,74^. Hence, both the compartmentalization of DA clusters in the fly brain and distinct post-synaptic effects exerted by different receptors within multiple neural substrates underlies the complex role of dopamine in regulating endogenous arousal (wakefulness) and exogenous arousal (behavioral responsiveness to sensory stimuli).

While, the split-GAL4 based neuronal targeting helps identify the specific sources of DA involved in endogenous arousal behaviors like wakefulness, the post-synaptic effects are more complex to pin down. All four dopamine receptors are co-expressed at high levels in the specific populations of Kenyon Cells (KCs) and DopR1 and DopR2 are enriched in MBONs that form the γ5, β′2 and γ4 MB compartments^75–78^.

**Figure 5:**
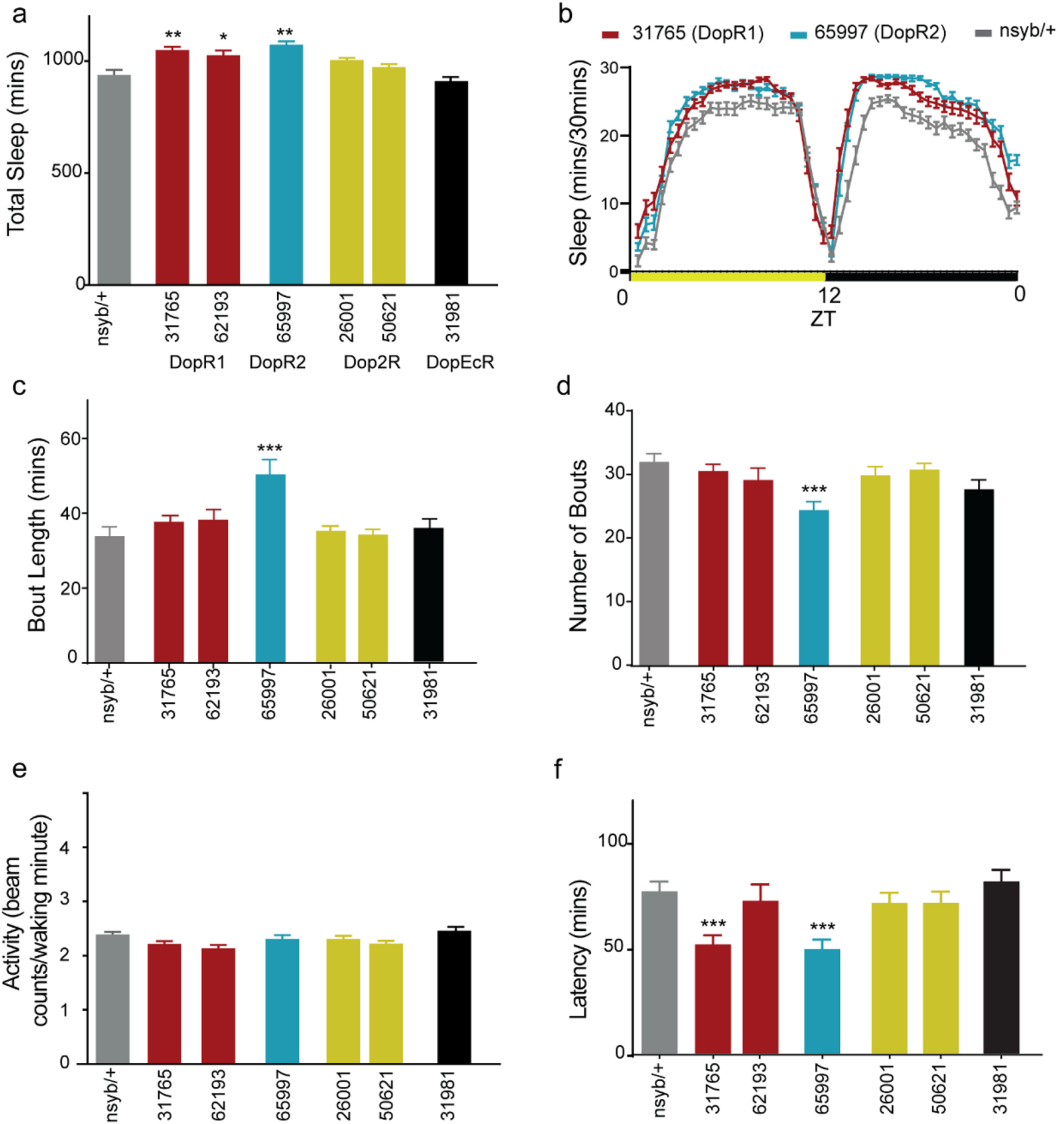
Pan-neuronal knockdown of dopamine receptors DopR1 and DopR2 increases sleep amount without altering waking activity. (**a**) Total sleep or sleep duration over 24 h in flies expressing validated UAS-RNAi lines targeting dopamine receptors specifically: DopR1 (31765, and 62193-red), DopR2 (65997-blue), Dop2R (2 lines: 26001 and 50621-yellow) and DopEcR (31981-black) pan-neuronally using nsyb-GAL4. Sleep data represents 2-day average (Day 3 and 4) at 24^ο^C after 2-day entrainment. nsyb-GAL4/+ flies (grey bars) were used as a negative control. (**b**) Representative sleep profile of RNAi mediated depletion of DopR1 (red) and DopR2 (blue) receptors in nsyb-GAL4 and control nsyb/+ (grey). ZT indicates zeitgeber time where ZT 0: lights on and ZT 12: lights off. (**c**,**d**) Average bout length and number of bouts. (**e**) Activity or average beam crossings/waking minute indicative of locomotor activity of all tested genotypes. (**f**) Sleep latency or time to sleep was calculated as the time gap between lights off and first sleep bout. Latency was reduced in nsyb/DopR1-RNAi (31765) and nsyb/DopR2-RNAi 65997) flies as compared to nsyb/+. For each of the experimental groups tested we had 88–98 flies which represented 4 independent experimental trials, sample included nsyb/+ (95), nsyb/31765 (96), nsyb/62193 (98), nsyb/65997 (93), nsyb/26001 (96), nsyb/50621 (96), and nsyb/31981 (88). Data represents mean and SEM, * indicates p < 0.05, ** indicates p < 0.001 and *** indicates p < 0.0001. Statistical analysis was one-way ANOVA and Dunnett’s paired comparison with control for (**a**,**e**,**f**) and Kruskal–Wallis non-parametric one-way ANOVA and Dunn’s post-hoc correction for (**c**,**d**).

We used a pan-neuronal driver nsyb-GAL4 (R57C10) with dicer expression and targeted all four dopamine receptors using validated UAS-RNAi lines in the context of sleep regulation. Given the wide variety of UAS-RNAi lines available to downregulate receptor transcripts we picked transgenic lines for each receptor that have been previously validated by quantitative RT PCR^39,40,46,54,79^. Flies with receptor knockdown were tested and we found that downregulation of DopR1 (two RNAi lines: 31765 and 62193) and DopR2 (one RNAi line: 65997) specifically increased total sleep without altering the locomotor activity measured by beam crossings/minute during wake-period (Fig. 5a,e).

Bout length and number of bouts were consistent between tested genotypes with the exception of one RNAi line targeting DopR2 (Fig. 5b–d). We also measured if latency is affected by manipulation of these receptors and found both DopR1 and DopR2 decrease latency even though it is not consistent between two UAS-RNAi lines targeting the DopR1 receptor (Fig. 5f). Taken together, pan-neuronal knockdown of DopR1 and DopR2 increased sleep and decreased sleep latency consistent with the role of DAN signaling in the MB.

### DopR1 and DopR2 regulate sleep amount, bout characteristics and sleep latency by influencing specific MB compartments

Since, pan-neuronal manipulations affect receptor levels outside of MB we repeated these experiments with validated RNAi lines that target DopR1 (31765 and 62193) and DopR2 (65997) and increase sleep. Although the lack of RNAi phenotypes for D2R and DopEcR does not rule the role of these receptors in sleep we focussed on DopR1 and DopR2 because previous experiments shows that P2X2 mediated activation of PAM DANs causes an increase in GCamp6m based fluorescence signal that is blocked by SCH23390, an antagonist of DopR1 and DopR2^32^.

We targeted DopR1 and DopR2 knockdown to MB neuronal populations that are potentially downstream to the to wake-active γ5, β′2 and γ4 PAM DANs using highly specific split-GAL4 lines described in^36,52^. All the RNAi lines used were inserted in the same genomic location on the 3rd chromosome for comparable expression. Specifically, we targeted two MB output neurons (MBONs) projecting to the γ5 (MB011B), β′2 (MB011B) and γ4 (MB298B) synaptic compartments and Kenyon cell populations projecting to these lobes (MB010B- all KCs, MB107B- α′β′ KCs).

**Figure 6:**
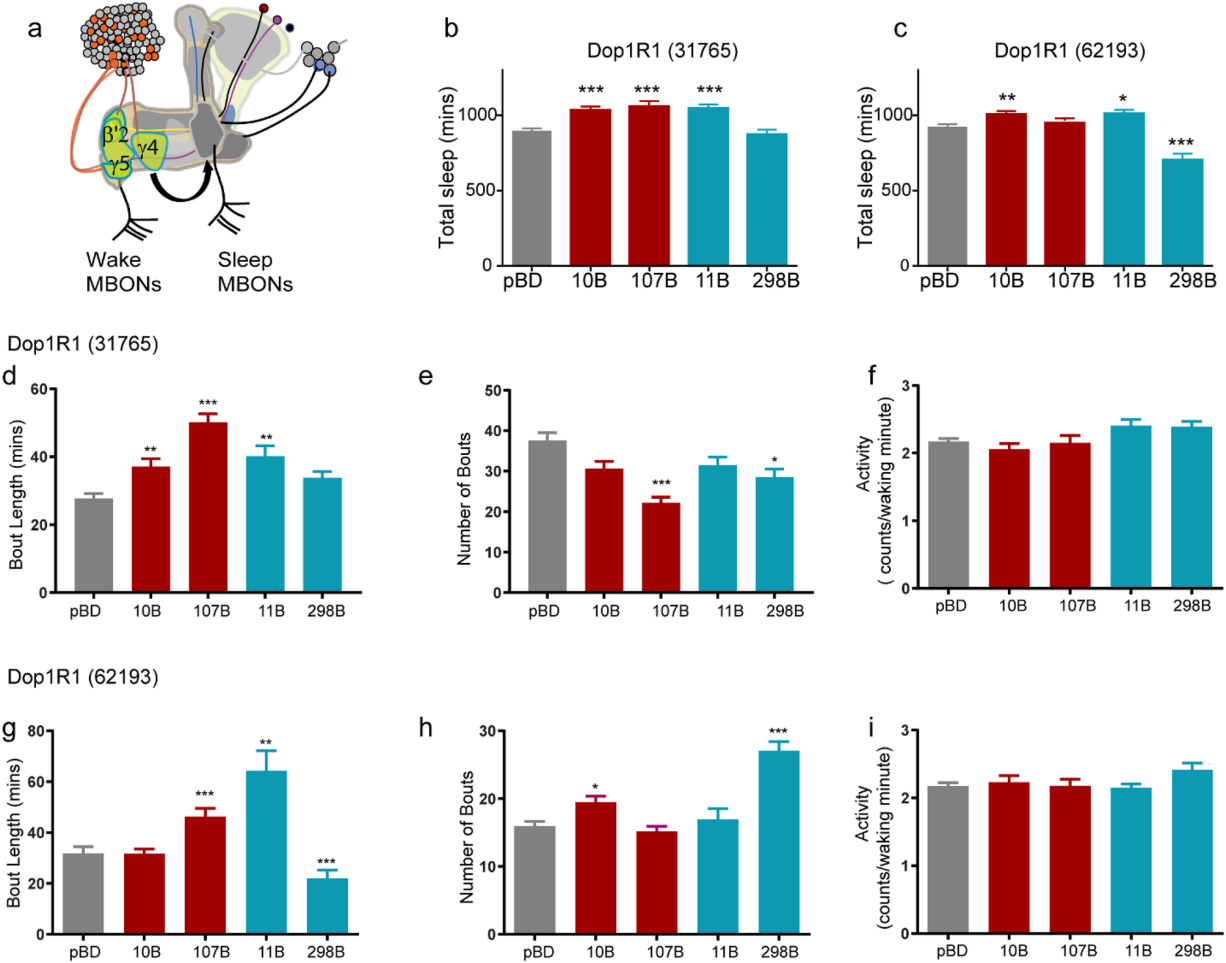
Knockdown of DopR1 in MB-KCs and MBONs of the sleep-regulating γ5 and β′2 compartment increased sleep. (**a**) Schematic of MB wake-promoting γ5, γ4 and β′2 compartments that represents the interactions between KCs, MBONs and PAM-DANs. (**b**,**c**) Total Sleep during a 24-h period in flies expressing UAS-DopR1 RNAi (31765 and 62193) in MB-KCs (MB010B, MB107B: red), MBONs (MB011B, MB 298B: blue) and enhancerless/empty split-GAL4 pBD (control: grey). (**d**,**e**) Average bout length and number of bouts in flies expressing UAS-DopR1 RNAi (31765) in MB-KCs (MB010B, MB107B: red), MBONs (MB011B, MB298B: blue) and enhancerless/empty split-GAL4 pBD (control: grey). (**f**) Activity or average beam crossings/waking minute indicative of locomotor activity of flies expressing UAS-DopR1 (31765). (**g**,**h**) Average bout length and number of bouts in flies expressing UAS-DopR1 RNAi (62193) in MB-KCs (MB010B, MB107B: red), MBONs (MB011B, MB298B: blue) and enhancerless/empty split-GAL4 pBD (control: grey). (**i**) Activity or average beam crossings/waking minute indicative of locomotor activity of flies expressing UAS-DopR1 RNAi (62193). For each of the experimental groups tested we had 37–48 flies which represented 2 independent experimental trials, sample included 31765/pBD (48), 31765/MB010B (40), 31765/MB107B (38), 31765/MB011B (38), 31765/MB298B (37), 62193/pBD (47), 62193/MB010B (39), 62193/MB107B (44), 62193/MB011B (43), and 62193/MB298B (47). Data represents mean and SEM, * indicates p < 0.05, ** indicates p < 0.001 and *** indicates p < 0.0001. Statistical analysis was one-way ANOVA and Dunnett’s paired comparison with control for (**b**,**c**,**f**,**i**) and Kruskal–Wallis non-parametric one-way ANOVA and Dunn’s post-hoc correction for (**d**,**e**,**g**,**h**).

One of the RNAi lines targeting DopR1 transcripts (31765) increased total sleep when expressed in γ5 β′2 MBONs (MBON 01, 03 and 04), α′β′ KCs and all KCs but not in γ4 MBONs as compared to pBD (control) suggesting that suppression of dopamine signaling via this receptor subtype increases total sleep (Fig. 6b). A closer analysis of the sleep structure reveals average bout length was higher in MB010B, MB107B and MB011B (Fig. 6d,e). Activity was consistent between tested genotypes showing that modulating receptor levels did not affect locomotor activity (Fig. 6f)^80^.

A second RNAi line targeting the same receptor (DopR1) increased total sleep when expressed in all KCs (MB010B) and γ5 β′2 (amp) MBONs (MB011B) without altering total sleep in α′β′ KCs (MB107B) and reducing sleep in γ4 MBONs (MB298B) (Fig. 6c). Like, the first DopR1 RNAi line (31765), increase in sleep was accompanied by increase in length of average sleep bout in MB107B and MB011B (Fig. 6g). Sleep bout number was mostly consistent between genotypes except for a small increase in MB010B which labels all KCs (Fig. 6h). Activity levels were consistent between genotypes (Fig. 6i).

In summary, two transgenes encoding RNAi lines targeting DopR1 showed consistent increase in total sleep and increased sleep bout length when expressed in MBON γ5 β′2, α′β′ KCs and all KCs. However, the effects on γ4 MBONs (MB298B) are perplexing as it reduces or has no effect on total sleep and sleep bout length.

**Figure 7:**
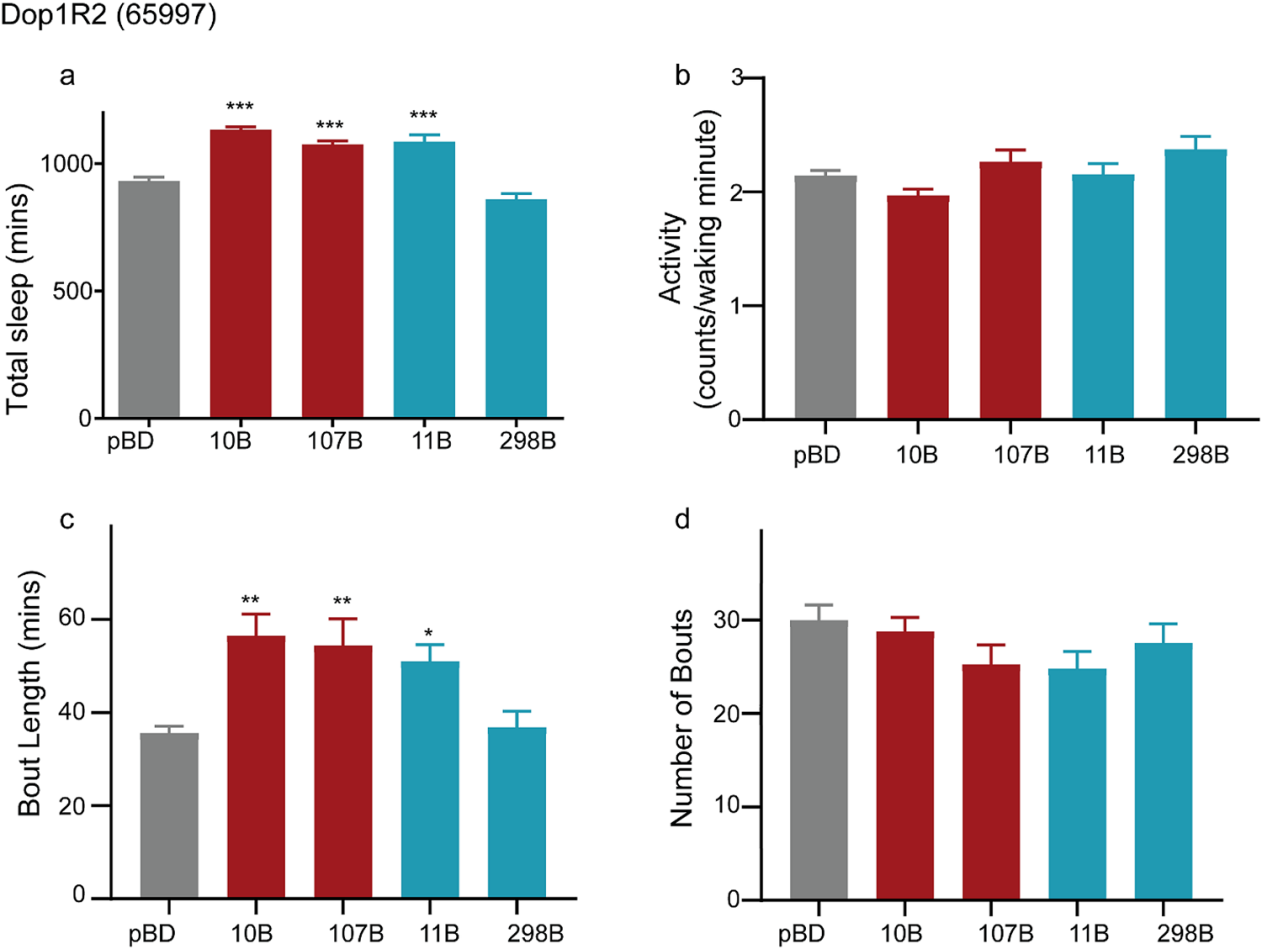
Knockdown of DopR2 in MB-KCs and MBONs of the sleep-regulating γ5 and β′2 compartment increased sleep. (**a**) Total Sleep during a 24-h period in flies expressing UAS-DopR2 RNAi (65997) in MB-KCs (MB010B, MB107B: red), MBONs (MB011B, MB 298B: blue) and enhancerless/empty split-GAL4 pBD (control: grey). (**b**) Activity or average beam crossings/waking minute indicative of locomotor activity of flies of all tested genotypes. (**c**,**d**) Average bout length and number of bouts in flies expressing UAS-DopR2 RNAi (65997) in MB-KCs (MB010B, MB107B: red), MBONs (MB011B, MB298B: blue) and enhancerless/empty split-GAL4 pBD (control: grey). For each of the experimental groups tested we had 44–48 flies which represented 2 independent experimental trials, sample included 65997/pBD (48), 65997/MB010B (46), 65997/MB107B (47), 65997/MB011B (44), and 65997/MB298B (48). Data represents mean and SEM, * indicates p < 0.05, ** indicates p < 0.001 and *** indicates p < 0.0001. Statistical analysis was one-way ANOVA and Dunnett’s paired comparison with control for (**b**,**c**,**f**,**i**) and Kruskal–Wallis non-parametric one-way ANOVA and Dunn’s post-hoc correction for (**d**,**e**,**g**,**h**).

Using, the above cell type specific regulation of DA receptors we downregulated the second D1 receptor, DopR2 receptor function (65997) in wake-regulating MBON compartments and Kenyon cell populations. We found that reduction in DA signaling via DopR2 receptor increased total sleep when expressed in γ5 (MB011B), β′2 (MB011B) and Kenyon cell populations projecting to these lobes (MB010B-all KCs, MB107B-α′β′ KCs) but not in γ4 (MB298B) MBONs (Fig. 7a).

This differential effect of DopR2 knockdown on MB011B and MB298B was similar to that observed for DopR1 (Fig. 6). In addition to total sleep, we also found that average length of sleep bout (Fig. 7c) was higher in MB010B, MB107B and MB011B as compared to MB 298B and empty-pBD negative control. Like DopR1, number of sleep bouts (Fig. 7d) and activity (Fig. 7b) was consistent between genotypes.

Taken together, these results show that PAM dopamine signaling to specific MB compartments requires both DopR1 and DopR2 receptor signaling specifically within the wake-regulating KCs or γ5 β′2 MBONs (MBON 01,03 and 04) or both but not γ4 (MBON 05) compartment.

### PAM γ5 signal via DopR1 and DopR2 to regulate total sleep and latency

While, UAS-RNAi transgene-induced gene silencing allows a spatial control, the efficacy of these transgenes and off-target effects are difficult to resolve in determining a clear role for DopR1 and DopR2 receptors in MB mediated sleep regulation.

**Figure 8:**
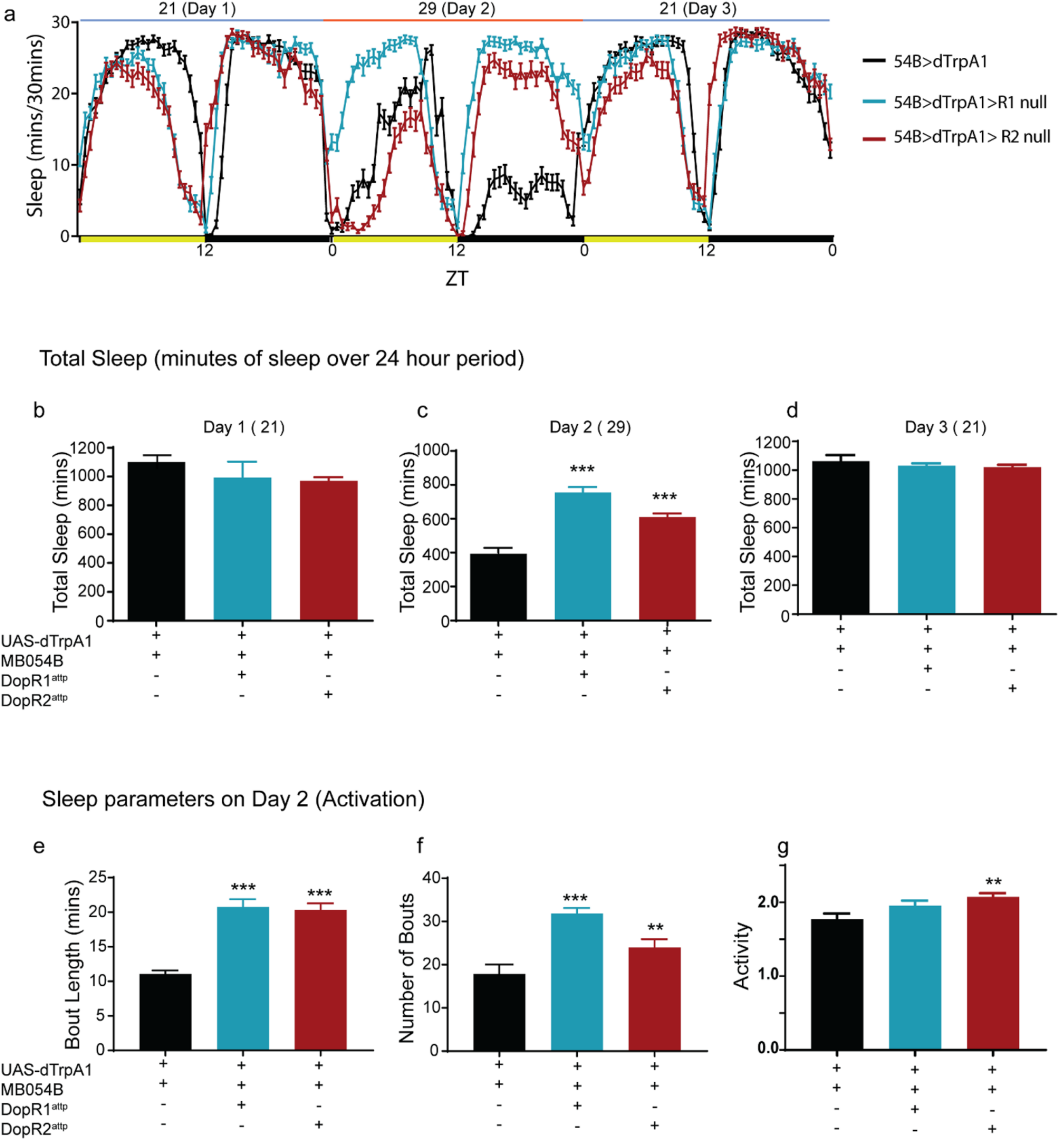
Wakefulness induced by PAM γ5 dopamine activation requires both DopR1 and DopR2 function. (**a**) 3-day Sleep profile of flies expressing temperature sensitive cation channel dTrpA1 in PAM γ5 DANs (MB054B) in the wild type, DopR1 and DopR2 hypomorph backgrounds. Day 1 represents baseline at 21 °C, Day 2 represent activation at 29 °C and Day 3 represents recovery at 21 °C. (**b**–**d**) Total sleep during Day 1 (baseline, 21 °C), Day 2 (activation, 29 °C) and Day 3 (recovery, 21 °C), of flies expressing dTrpA1 in PAM γ5 in positive control (w^1118^) and receptor hypomorph background (DopR1^attp^ and DopR2^attp^). (**e**,**f**) Average sleep bout duration and number of bouts on Day 2 (activation, 29 °C) of flies expressing dTrpA1 in PAM γ5 in positive control (w^1118^) and receptor hypomorph background (DopR1^attp^ and DopR2^attp^). (**g**) Activity or average beam crossings/waking minute, a measure of locomotor activity for all tested genotypes. For each of the experimental groups tested we had 59–61 flies which represented 2 independent experimental trials, sample included MB054B > dTrpA1 (59), MB054B > dTrpA1 > DopR1^attp^ (59) and MB054B > dTrpA1 > DopR2^attp^ (61). Data represents mean and SEM, * indicates p < 0.05, ** indicates p < 0.001 and *** indicates p < 0.0001. Statistical analysis was one-way ANOVA and Dunnett’s paired comparison with control for (**b**,**c**,**d**,**g**) and Kruskal–Wallis non-parametric one-way ANOVA and Dunn’s post-hoc correction for (**e**,**f**).

In order to directly address and test the coordinated role of PAM γ5 signaling through DopR1 and DopR2 receptor in wake regulation we specifically activated PAM γ5 (MB054B) neurons using a temperature sensitive cation channel dTrpA1 in DopR1 and DopR2 hypomorph backgrounds^81,82^. We measured total sleep in flies at 21 °C (permissive temperature), the day before activation (baseline, Fig. 8a) during which dTrpA1 channels expressed in PAM γ5 neurons are closed in w^1118^, DopR1, and DopR2 hypomorph background. We did not find any significant differences between the three tested genotypes during baseline (Fig. 8b).

However, at 29 °C when PAM neurons are activated in w^1118^ background we find significant decreases in sleep (from ~ 1000 min at baseline to ~ 400 min on day 2, Fig. 8b). Sleep suppression as a result of PAM activation affects both daytime and nighttime sleep (Fig. 8a) even though the effects are stronger during nighttime. The sleep suppression caused by PAM γ5 activation was blocked in the DopR1 and DopR2 mutant background (Fig. 8c). These effects were reversible and total sleep is consistent between genotypes (Fig. 8d), when temperature is switched back to 21 °C (permissive temperature, Day 3), at which dTrpA1 channel is no longer open.

The wakefulness induced by activation of MB054B PAM DANs significantly reduces the bout length and bout number (Fig. 8e,f). Both of these effects were blocked in the DopR1 and DopR2 mutant background suggesting that these receptors are required for regulation of sleep duration and bout structure by PAM DANs. Locomotor activity was consistent between genotypes (Fig. 8g).

## Discussion

The mushroom body lobes are tiled by discrete anatomic compartments defined by the axons of a specific subset of DANs and the dendrites of one or two mushroom body output neurons (MBONs). This anatomical arrangement positions DANs to strategically convey positive and negative reinforced information by changing the synaptic weight of KC-MBONs in producing aversive and appetitive responses^36,52^.

While, the most in-depth analysis of these synapses and distinct DAN-KC-MBON connectivity and behavioral output comes from studies of olfactory conditioning, there is evidence that these synapses play a critical role in innate behaviors like feeding and sleep^32,40^. Although, role of DA on sleep has been extensively investigated in *Drosophila*, the commonly used TH-Gal4 driver line labels most dopamine neuron clusters, but is absent from the several PAM clusters that projects to MB^83^.

In this study we specifically probed PAM subsets that project to γ5, γ4, and β′2 MB compartments. We focused on this subset because KCs and MBONs downstream of these PAM neurons can be neuroanatomically resolved and have been shown to be required for wakefulness. Further, KCs and MBONs that form the γ5, γ4, and β′2 synaptic compartments alter their spontaneous neural activity in response to sleep need (induced by mechanical sleep-deprivation)^31^. The ability to use cell-specific split-GAL4 tools provides opportunity to resolve the precise circuit mechanisms by which PAM neurons regulate wakefulness.

GABA signaling also modulates sleep and wake microcircuits within MB^53^. The key source of GABA in the MB is anterior paired lateral neurons, APL and dorsal paired medial neurons (DPM), which are electrically coupled and increase sleep by GABAergic inhibition of wake-promoting KCs^53^. In the context of associative learning, there is strong evidence for interactions between KCs, APL, DPM and DANs^54,55^ but it is not clear if GABA and dopamine signaling represent opposing inputs to the KCs and MBONs in the regulation of sleep. Here, we find that the excitability of PAM DANs involved in wakefulness is blocked by sleep-promoting GABA signaling and mediated by ionotropic receptor subtype GABA_A_-Rdl.

A recent study showed that GABA inhibitory input to the presynaptic terminals of the PAM neurons regulates appetitive memory and that this interaction is mediated by GABA-B3 receptors that are clustered in PAM boutons localized to PAM-γ5 and -α1 compartments^84^. These data are consistent with our findings that PAM-γ5 are GABA responsive and that multiple receptors are critical to this interaction. Since, we did not find a role for GABA-B3 in PAM mediated sleep regulation, it is likely that PAM γ5, γ4, and β′2 express multiple GABA receptors which are differentially recruited in sleep and learning. How and what regulates the expression of these receptors in PAM subsets presents a potential mechanism of presynaptic gating to MB core circuits. Transcriptomic analysis of PAM neurons reveals extremely high levels of Rdl expression followed by GABA-B3. Among the PAM subsets mean TPM or transcripts per million of Rdl receptor in PAM γ5, γ4, and β′2 are much higher as compared to other PAM subsets^49^.

Simple connection query search of the recently released hemibrain data^85^ reveals there is significant bidirectional connectivity between APL, DPM, and PAM neurons (neuprint.janelia.org). Further, a recent study showed that APL neurons express the inhibitory D2R receptor^55^. APL mediated GABAergic inhibition of the PAM neurons was recently shown to control the intensity and specificity of olfactory appetitive memory but previous results show that blocking GABA release from APL neurons only modestly affects sleep phenotypes^53,84^.

While, the role of APL in GABA signaling to PAM γ5, γ4, and β′2 cannot be completely ruled out, other inputs to wake-regulating PAM DANs could also be GABAergic and critical for promoting sleep. A recent study using EM dataset of a Full Adult Female Fly Brain (FAFB) mapped the inputs and outputs of the PAMγ5 DANs and identified that this cell type is highly heterogenous and in addition to recurrent feedback from MBON01 γ5β′2a, it receives extensive input from other MBONs, sub-esophageal output neurons (SEZONs) and lateral horn output neurons^86^. The EM data also reveals that octopaminergic neurons synapse onto PAM γ5, γ4, and β′2 DANs. Whether, these inputs play a role in wakefulness is unknown but suggests that the PAMγ5 could serve as a key link between sensory inputs, wake-promoting octopamine signal and core sleep regulating circuitry within the MB. Each of these inputs could modulate PAM-DAN activity and dopamine release in regulating wakefulness via the MB.

In addition to probing the release and activity of these PAM-DANs we also explored the dopamine receptors and their location within the MB in signaling wakefulness. To this end we expressed validated RNAi lines in subsets of KCs and MBONs and find that DopR1 and DopR2 are critical in mediating the wakefulness signal via KCs and γ5β′2 MBONs. Knocking down the receptor consistently increased total sleep and bout length. Furthermore, specific manipulations of DopR receptors within the MB did not directly alter locomotor activity as observed by manipulation of these receptors in CX^80^. Although, loss-of-function mutations of D1 dopamine receptor DopR are shown to enhance repetitive air puff startle-induced arousal and increase sleep. Expression and restoration of DopR in the mutant background specifically in the central complex rescues the startle response, while, the sleep phenotype is rescued via a broad MB driver^87^. Our data extends these findings by showing that the DopR receptors regulate sleep via the MB γ5 and β′2 compartment. Although, targeted RNAi experiments show that DopR’s are required for sleep regulation by KCs and MBONs, the lack of a sleep phenotype in DopR2 mutant could be a result of global loss of receptor in the mutant as opposed to targeted loss of receptor function within MB. Dopamine signals wakefulness by activation of wake-promoting neurons of MB via DopR1 and DopR2 and within. the central complex, neurons of dFB are inhibited by dopamine via DopR2^26^. Hence, DopR2 has opposing effects within MB and CX.

In vitro characterization indicates that DopR’s signal through distinct G-proteins, with DopR1 via Gαs to stimulate cAMP production^72,88^ and DopR2 coupling to Gαq via increased calcium^71,77^. These receptors are thought to have differential sensitivity to dopamine^77^ and could be potentially recruited by varying DA release or DAN activity. In the context of sleep regulation, our work reveals that both DopR1 and DopR2 induce wakefulness via the γ5 β′2 MB compartment but not γ4 compartment. Although, chronic activation of PAM γ4 induces wakefulness, the glutamatergic MBON γ4 < γ1,2 projects to multiple compartments and could potentially activate or inhibit MBONs and PAMs projecting to γ1 and γ2 compartment. The interaction between compartments is not well understood in the context of sleep and wake regulation and requires further investigation to better understand the role of DopR2 in regulating the γ4 compartment. The neuroanatomical specificity obtained from split-Gal4 lines combined with EM data has paved way for more detailed analysis of the role of dopamine signaling to MB in the context of sleep and other behaviors.

The sleep-regulating PAM DANs and associated KCs and MBONs identified in our study are also involved in mediating satiety, novelty, caffeine induced arousal, punishment and reward associated experiences suggesting that the activity of these neurons is tuned to several wake and arousal associated behaviors^35,43,49,89–92^. This is further supported by the EM connectome data showing that MB receives extensive gustatory, auditory and visual input in addition to olfactory input^93^.

Current models of sleep regulation rely on two main processes, the circadian clock and the sleep homeostat and don’t completely account for multiple external and internal factors that influence wakefulness^94^. The ability to sleep, however, is influenced by motivational or cognitive stimuli. We therefore envision that sleep, wakefulness and arousal within MB are not located in distinct circuits, but rather mediated by distinct processes within a common circuit.

## Methods

### Fly stocks and rearing conditions

All Fly stocks were maintained on cornmeal-agar-molasses medium (https://bdsc.indiana.edu/information/recipes/molassesfood.html) in 12 h light: 12 h dark conditions at 18 °C with ambient humidity of 60–70%. The light intensity in the incubator was between 500 and 1200 lx measured using a luxmeter (Dr. Meter 1330B-V Digital Illuminance/Light Meter 0–200,000 Lux, Amazon Inc). Rearing and manipulation including virgin collection, genetic crosses and progeny collection for behavioral experiments was carried out in cornmeal dextrose agar media (https://bdsc.indiana.edu/information/recipes/dextrosefood.html). Age matched flies (3–7-day old) were collected and used for behavioral experiments, and immunohistochemistry. The following stocks used in the experiments were obtained from Bloomington Drosophila Resource Center:

- 24651—w[1118]; P{w[+ mC] = UAS-Dcr-2.D}10
- 26263—w[*]; P{y[+ t7.7] w[+ mC] = UAS-TrpA1(B).K} attP16
- 27699—y[1] v[1]; P{y[+ t7.7] v[+ t1.8] = TRiP.JF02779}attP2
- 28353—y[1] v[1]; P{y[+ t7.7] v[+ t1.8] = TRiP.JF02989}attP2/TM3, Sb[1]
- 26729—y[1] v[1]; P{y[+ t7.7] v[+ t1.8] = TRiP.JF02271}attP2
- 89903—w[1118]; P{w[+ mC] = UAS-Rdl.RNAi.8–10}G
- 89904—w[1118]; P{w[+ mC] = UAS-Rdl.RNAi.4–5}E
- 31765—y[1] v[1]; P{y[+ t7.7] v[+ t1.8] = TRiP.HM04077} attP2
- 55239—y[1] sc[*] v[1]; P{y[+ t7.7] v[+ t1.8] = TRiP.HMC02344} attP2/TM3, Sb[1]
- 62193—y[1] sc[*] v[1]; P{y[+ t7.7] v[+ t1.8] = TRiP.HMC05200} attP40
- 26018—y[1] v[1]; P{y[+ t7.7] v[+ t1.8] = TRiP.JF02043} attP2
- 51423—y[1] sc[*] v[1]; P{y[+ t7.7] v[+ t1.8] = TRiP.HMC02893} attP2
- 65997—y[1] sc[*] v[1]; P{y[+ t7.7] v[+ t1.8] = TRiP.HMC06293} attP2
- 26001—y[1] v[1]; P{y[+ t7.7] v[+ t1.8] = TRiP.JF02025} attP2
- 36824—y[1] sc[*] v[1]; P{y[+ t7.7] v[+ t1.8] = TRiP.GL01057} attP2
- 50621—y[1] v[1]; P{y[+ t7.7] v[+ t1.8] = TRiP.HMC02988} attP40
- 50622—y[1] v[1]; P{y[+ t7.7] v[+ t1.8] = TRiP.HMC02989}attP40
- 31981—y[1] v[1]; P{y[+ t7.7] v[+ t1.8] = TRiP.JF03415} attP2
- 32194—w[*]; P{y[+ t7.7] w[+ mC] = 20XUAS-IVS-mCD8::GFP} attP2
- 39171—w^1118^; P{GMR57C10-GAL4} attP2
- 42748—w^1118^; P{y[+ t7.7] w[+ mC] = 20XUAS-IVS-GCaMP6m} attP40
- 91222—y[1] w[*]; P{w[+ mC] = UAS-P2X2.L}3
- 91223—w[*]; P{w[+ mC] = UAS-Rnor\P2rx2.L}4/CyO
- 41347—w[1118]; P{y[+ t7.7] w[+ mC] = GMR58E02-GAL4}attP2

Split-GAL4 lines: MB054B, MB312B, MB196B, MB194B, MB213B, MB209B, MB060B, MB011B, MB010B, MB107B, MB298B, and pBDGAL4 were obtained from Dr. Yoshinori Aso and Dr. Gerry Rubin and have been described in^32,35,36,52^. For experiments using GAL4 lines, we used BDPGAL4U as a negative control, which contains the vector backbone used to generate each GAL4 line, but lacks any active enhancer motif to drive GAL4 expression^95,96^. Deletion mutants DopR1 (DopR1^attp^) and DopR2 (DopR2^attp^) were obtained from Todd Laverty and described in^82^. In both these deletion lines, the first coding exon has been deleted and replaced by an attP site.

### Sleep assays

For sleep experiments males and females were collected 3–7 days post-eclosion and placed in 65 mm × 5 mm transparent plastic tubes with standard cornmeal dextrose agar media, placed in a Drosophila Activity Monitoring system (Trikinetics Inc.), and locomotor activity data were collected in 1 min bins. Activity monitors were maintained in a 12 h:12 h light–dark cycle at 65% relative humidity, and flies were given 48 h to acclimate and entrain to the light/dark cycle of the incubator. Total 24-h sleep quantity for each day of the experiment was extracted from locomotor activity data and sleep is defined as a contiguous period of inactivity lasting 5 min or more^97^. Sleep profiles were generated depicting average sleep (minutes per 30 min) for the days of the experiment and maintained in the same tube. For CBZ experiments flies were placed on drug food the day prior to Shi^ts1^ inhibition as indicated in the experimental schematics in Figs. 3 and S2.

All dTrpA1^81^ and Shi^ts1^^98^ experiments were conducted using temperature shift of 21 °C (permissive) and 29 °C (restrictive) and RNAi experiments were conducted at 24 °C. For RNAi experiments data represents an average of 2 days post-entrainment. For temperature shift experiments permissive temperature controls and genotypic controls were used for hit detection as indicated. Data analysis for sleep experiments was performed using MATLAB-based software SCAMP developed by Dr. Christopher Vecsey (Skidmore College) and an earlier version of the software was published in^99^. For all screen hits, waking activity was calculated as the number of beam crossings/min when the fly was awake. Statistical comparisons between experimental and control genotypes were performed using Prism 7 (GraphPad Inc, CA).

### Carbamazepine feeding

CBZ (Sigma-Aldrich, C4024**)** was dissolved in 45% (2-hydroxypropyl)-beta-cyclodextrin (Sigma-Aldrich, H107) as described in to prepare a stock solution^100^. For CBZ experiments, flies were loaded in tubes containing 2% agarose (A9539, Sigma) and 5% sucrose (S0389, Sigma-Aldrich) with 0.1 mg/ml CBZ.

### Calcium imaging experiments

Transgenic flies expressing UAS-P2X2 and UAS-GCamp6m were dissected in hemolymph-like HL3 solution (5 mM HEPES pH 7.2, 70 mM NaCl, 5 mM KCl, 1.5 mM CaCl_2_, 20 mM MgCl_2_, 19 mM NahCO_3_, 5 mM trehalose, and 115 mM sucrose). Freshly dissected brains were placed on a poly-l-lysine coated cover glass in a recording chamber (PC-H chamber, Siskiyou Inc, OR) with HL3 solution. For GCamp6m based measurement, brains were equilibrated with HL3 or 50 mM GABA (0344, Tocris Inc) for 5 min prior to bath application of 5 mM ATP (A26209, Sigma Inc). A time series of fluorescence images was acquired using an Olympus BX51W microscope with U Plan Aprochromat 40X water immersion objective. GCamp6m was excited with a 470 nm LED light source (X-Cite turbo multiwavelength system) and images were acquired using ORCA FLASH 4.0 V2 digital CMOS camera. The average fluorescence of all pixels for each time point within a ROI was subtracted from the average background fluorescence of an identically sized ROI elsewhere within the brain as described in^31,32^. The resulting pixel fluorescence value for each time point was defined as Ft. Changes in fluorescence were calculated as ΔF/F = ((Ft − Fo)/Fo) where Fo is defined as the average background-subtracted baseline fluorescence for the 10 frames preceding ATP application. All images were processed and quantified using CellSens (Olympus Inc.) and Fiji (Image J).

### Immunohistochemistry

Dissection and immunohistochemistry of fly brains were performed as previously described with minor modifications (https://www.janelia.org/project-team/flylight/protocols). Brains of 3–7 day old male flies were dissected in 1X PBS medium (BP3920, Fisher Sci) and fixed in 2% paraformaldehyde (PFA, 15710 Electron Microscopy Sciences) in PBT for 60 min at room temperature (RT). After washing in PBT (0.5% Triton X-100 from Sigma X100 in PBS), brains were blocked in 5% normal goat serum (NGS) (S1000 Vector Laboratories) in PBT overnight. Brains were then incubated in primary antibodies in NGS, nutated for 4 h at room temperature, then transferred to 4 °C for 2 days, washed three times in PBT for 30 min, then incubated in secondary antibodies diluted in NGS, nutated for 4 h at room temperature, then transferred to 4 °C for 2 days. Brains were washed thoroughly in PBT three times for 30 min and mounted in Vectashield (H-1000, Vector laboratories, CA) for imaging. The following antibodies were used: rabbit anti-GFP (A11122, 1:1000; Invitrogen), mouse nc82 (1:50; Developmental Studies Hybridoma Bank, Univ. Iowa), and cross-adsorbed secondary antibodies to IgG (H + L): goat Alexa Fluor 488 anti-rabbit (A11034, 1:800; Invitrogen) and goat Alexa Fluor 568 (A11031, 1:400; Invitrogen).

### Statistical analysis

Different sleep parameters (sleep amount, activity, bout length and number of bouts) are presented as bar graphs and represent mean ± SEM. A one‐way ANOVA was used for comparisons between two or more treatments or two or more genotypes and post hoc analysis was performed using Dunnett’s correction. For data sets that did not follow a gaussian/normal distribution (bout numbers and bout length) we used non-parametric analysis (one-way ANOVA of ranks and Kruskal Wallis Statistic). For comparisons of calcium levels between genotypes or treatments we used t-tests (two-tailed). All statistical analyses and graphing were performed using Prism software (GraphPad Software 7.04; San Diego, California).

## Supplementary Information

Supplementary Information.
