# Supplementary material for: Compartment specific regulation of sleep by mushroom body requires GABA and dopaminergic signaling
Source: Sci Rep. 2021 Oct 8;11:20067. doi: 10.1038/s41598-021-99531-2 (PMC8501079; doi:10.1038/s41598-021-99531-2)
Supplement: Supplementary file 1 — Supplementary Information. [file 41598_2021_99531_MOESM1_ESM.docx]

**Supplementary Figures:**

**3 Supplementary Figures (S1, S2 and S3)**


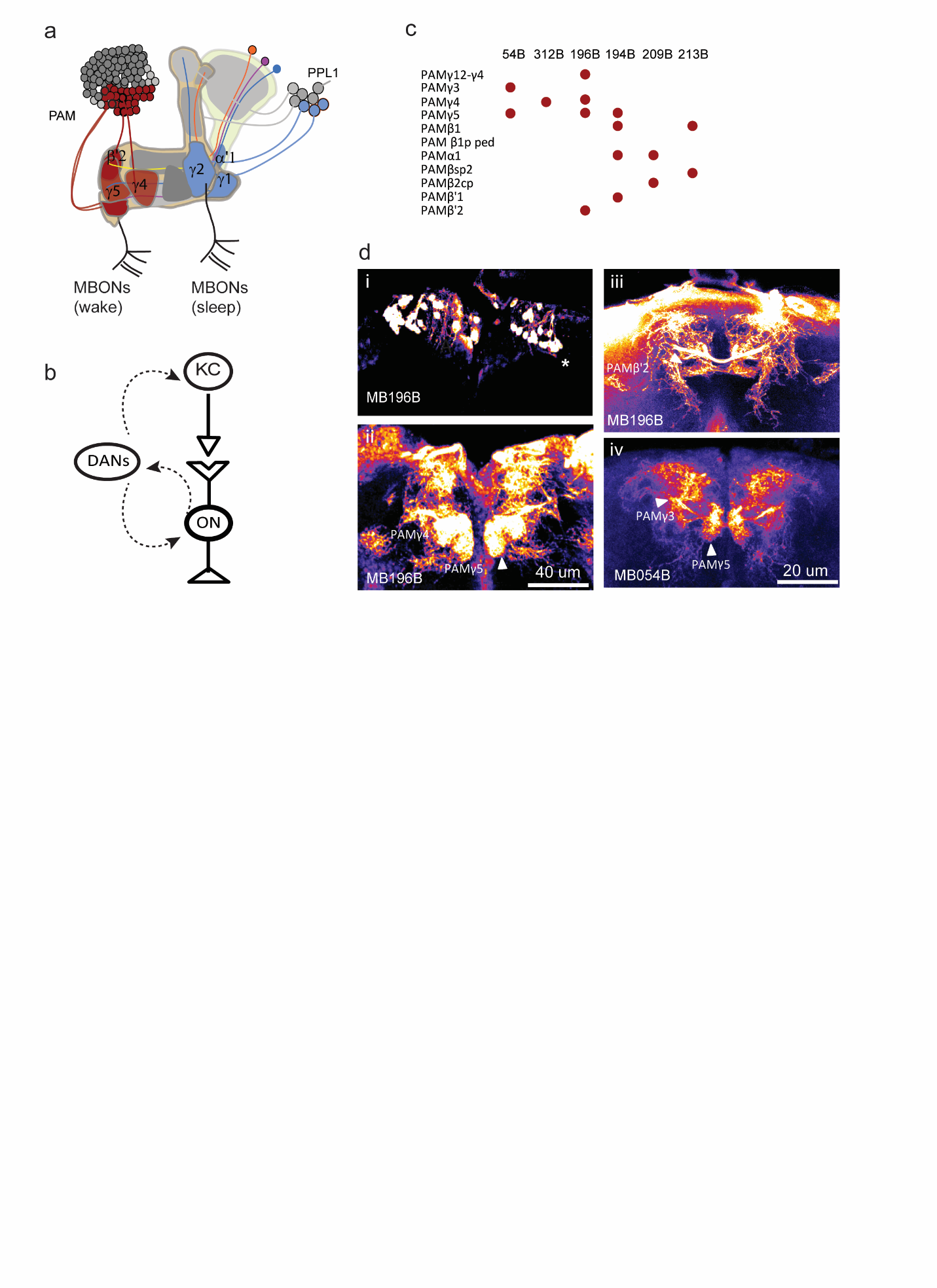


**Figure S1: Lobe specific innervations of wake-promoting PAM-DAN subsets.** The expression patterns with lobe specific innervation of these specific and broad sleep-regulating dopamine drivers were confirmed by expression of UAS-mCD8-GFP.

(a) Schematic representation of MB showing lobe specific axonal projections of PAM (red) and PPL1 (blue) DANs. Subset of PAM innervate the MB compartments (red) involved in regulating wakefulness mediated by KCs and MBONs.

(b) Schematic representation of synaptic interactions between PAM DANs, KCs and MBONs with MB compartments.

(c) Lobe specific projections of split-GAL4 driver lines used to label single or multiple subsets of PAM neurons innervating to the wake-promoting KCs and MBONs of MB.

(d) Maximum intensity projections of confocal stacks (10-15 slices) representing the MB regions in fire LUT applied to emphasize PAM (D i-iv) neurons. Cell bodies (asterisks) and lobe specific innervations (arrowhead) have been indicated to identify MB compartment innervated by each of the split-GAL4 used. Scale bars indicate 20um or 40um.


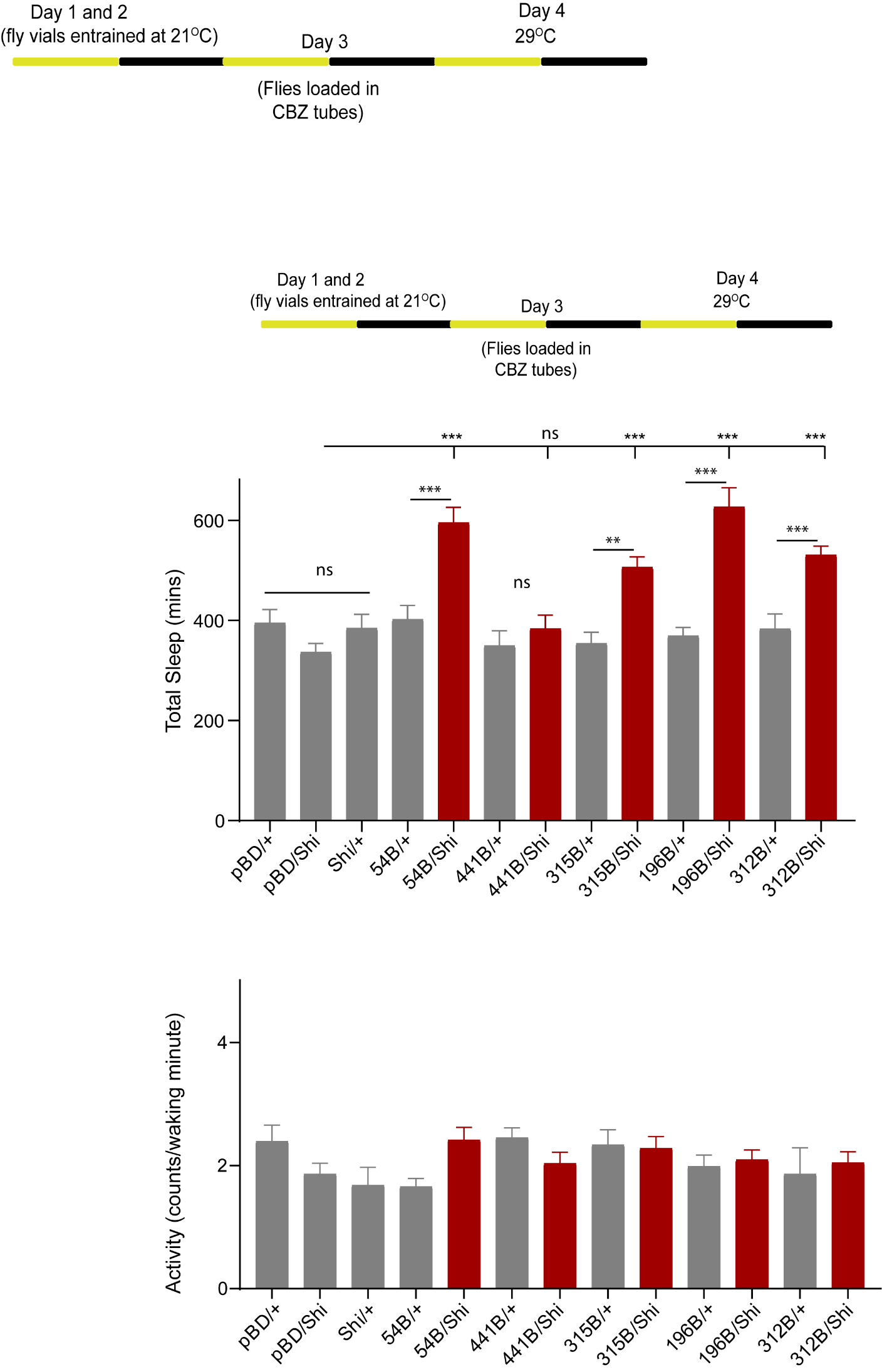


**Figure S2: Wakefulness induced by pharmacologic suppression of GABA_A_ receptor, Rdl requires PAM** **γ5 but not PAM γ3 neurons**. Schematic of experimental protocol showing temperature and drug conditions over a 5-day period. Flies were entrained for Day 1 and 2 in vials. Following entrainment flies were loaded on CBZ containing food (Day 3) in incubator maintained at 21^ο^C. Day 4 the temperature was switched to 29^ο^C and used for quantification and analysis shown below.

(a) Total sleep in PAM-DAN subsets (red) labelled by split-GAL4 lines MB054B, MB315B, MB441B, 312B and 196B where neural activity has been suppressed by over-expressing temperature sensitive dominant negative dynamin mutation, Shi^ts1^ in the presence of CBZ at 29^ο^C (Day 4). Enhancerless/empty-GAL4 in the same genetic background as PAM split-GAL4 lines (pBD/+, pBD/Shi) and split-Gal4/+ were used as additional genetic controls(grey).

(b) Activity or average beam crossings/waking minute indicative of locomotor activity of all tested genotypes.

For each of the experimental groups we had 34-51 flies which represents 2 independent experimental trials. Number of flies for each genotype were: pBD/Shi (n=51), shi/+ (n=50), 54B/+ (n=40), 441B/+ (n=38), 315b/+ (n=34), 54B/Shi (n=38), 441B/Shi (n=45) and 315B/Shi (n=35), 312B/+(39), 312B/Shi (46), 196B/+ (38) and 196B/Shi (36).

In this and all subsequent figures data represents mean and SEM, * indicates p<0.05, ** indicates p<0.001 and *** indicates p<0.0001. Statistical analysis was one-way ANOVA and Dunnett’s paired comparison with control (pBD/Shi) for A and B. We also ran pairwise comparisons between split-Gal4/+ and split-Gal4/Shi.


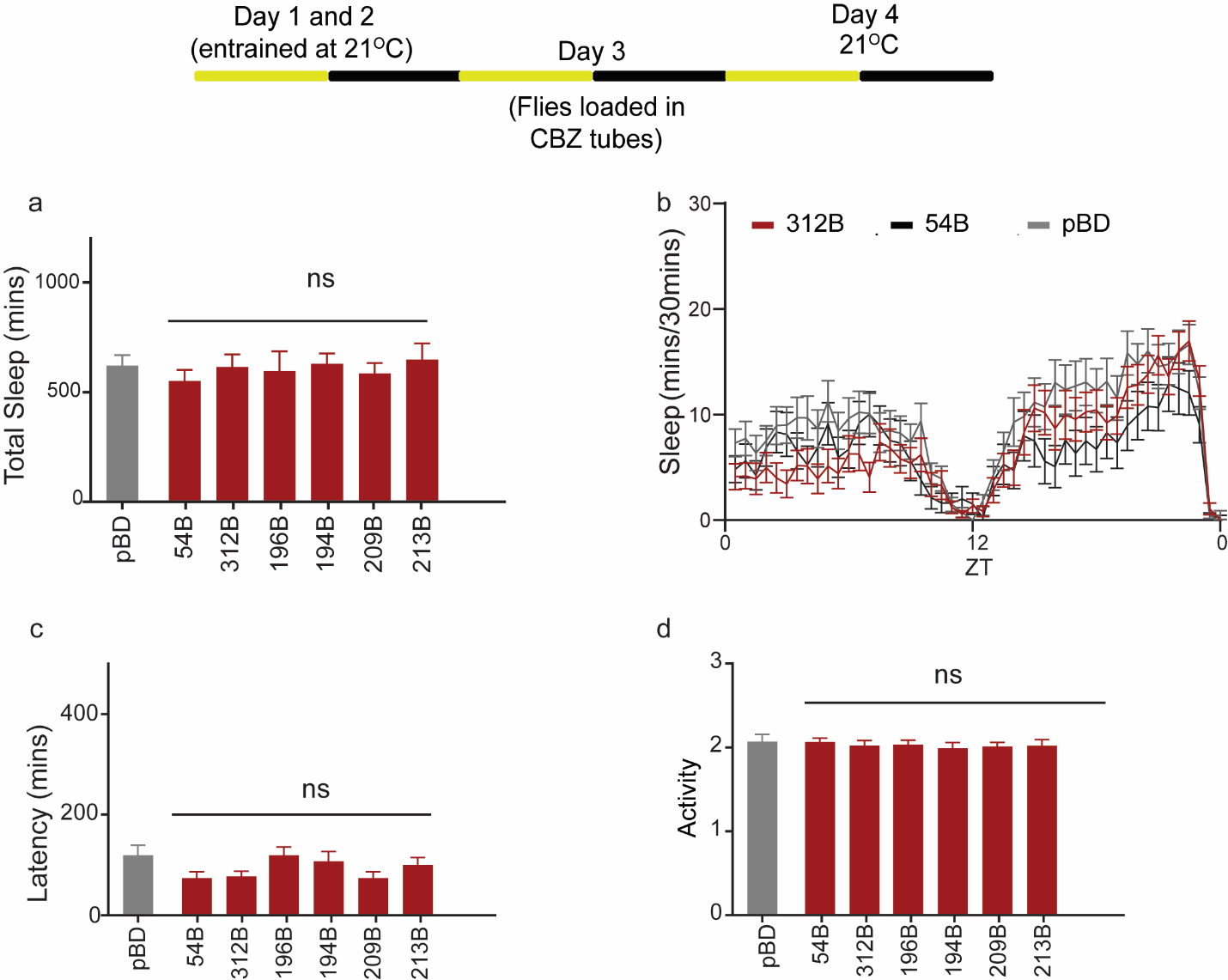


**Figure S3: In the absence of Shi^ts1^-mediated inhibition of PAM-DANs, all genotypes responded equivalently to CBZ.** Schematic of experimental protocol showing temperature and drug conditions over a 5-day period. Mated female flies were loaded on CBZ containing food (Day 3) in incubator maintained at 21^ο^C. Day 4 the temperature was maintained at 21^ο^C and used for quantification and analysis below.

(a) Total sleep in PAM-DAN subsets (red) labelled by split-GAL4 lines, MB054B, MB312B, MB194B, MB196B, MB209B, and MB213B where neural activity has been suppressed by over-expressing temperature sensitive dominant negative dynamin mutation, Shi^ts1^ in the presence of CBZ (Day 4). Enhancerless/empty-GAL4 in the same genetic background as PAM split-GAL4 lines was used as control (grey).

(b) Representative sleep profile of flies on Day 4 with targeted inhibition of specific PAM MB312B (red), MB054B (black) and empty/enhancerless-GAL4 control (grey). ZT indicates zeitgeber time where ZT 0: lights on and ZT 12: lights off.

(c) Sleep latency or time to sleep from lights off (ZT 12) was calculated as the time gap in minutes between lights off and first sleep bout.

(d) Activity or average beam crossings/waking minute indicative of locomotor activity of all tested genotypes.

For each of the experimental groups we had 38-51 flies which represents 2 independent experimental trials. Number of flies for each genotype were: pBD (n=45), 54B (n=46), 194B (n=45), 209B (n=45), 213B (n=38), 312B (n=40), and 196B (n=40). Data represents mean and SEM, * indicates p<0.05, ** indicates p<0.001 and *** indicates p<0.0001. Statistical analysis was one-way ANOVA and Dunnett’s paired comparison with control for (a, c and d).
